# Detection of Filtration Characteristics of Nontraditional Asymmetric Microporous Membranes Using Size-Controllable Micro-Hydrogel

**DOI:** 10.3390/polym17212958

**Published:** 2025-11-06

**Authors:** Hao Zhang, Tiantian Zhu, Yushan Zheng, Weiheng Liu, Tangxin Zhang, Yuhua Mao, Jiayuan Wang, Lingyu Zhu, Cheng Xu, Jianli Wang

**Affiliations:** State Key Laboratory of Advanced Separation Membrane Materials, State Key Laboratory of Green Chemical Synthesis and Conversion, Zhejiang Province Key Laboratory of Biofuel, College of Chemical Engineering, Zhejiang University of Technology, Hangzhou 310014, China; haozhang2019@zjut.edu.cn (H.Z.);

**Keywords:** microporous membrane, asymmetric pore structure, micro-hydrogel suspension

## Abstract

Microporous membranes are frequently used to remove or concentrate suspended solids. To maximize filtration efficiency for certain high-value liquids, a microporous membrane with a nontraditional asymmetric topology was recently developed to treat bio-based liquids, such as the isolation of proteins/enzymes from concentrates or the concentration of active cells from cultivation media. In this study, compared with both asymmetric and symmetric membranes, we reveal the unique filtration properties of “upper-stream open” asymmetric membrane using four types of fluids comprising monodispersed micro-hydrogels with sizes ranging from 294 to 517 nm. The results indicate that the internal pore structures of the membranes significantly affect the retention of microhydrogels of identical sizes. Asymmetric membranes offer considerable advantages in terms of retention efficiency and particle localization. By applying four classical blocking models along with adsorption models, the primary blockage mechanisms in asymmetric membranes for microgels of different sizes were explored. These results offer a better understanding of the interaction between the membrane and filtrate, assist in membrane selection, and elucidate the experimental results of membrane filtration.

## 1. Introduction

Suspension filtration technology plays a critical role in water treatment, pharmaceutical manufacturing, and public health [1,2,3,4]. Microfiltration (MF) and ultrafiltration (UF) technologies are widely applied in bioengineering through size exclusion and adsorption mechanisms, owing to their low energy consumption and high retention efficiency. The average pore size and porosity of porous membranes are key factors that determine the filtration performance of MF/UF [5,6,7,8]. In addition, the polarity and charge on the surface of the membranes play important roles in the capture of the suspension [9]. Lee et al. introduced negatively charged functional groups onto the surface of a polyvinylidene fluoride membrane, which generated electrostatic repulsion against negatively charged bacteria and thus enhanced bacterial rejection [10]. Moreover, the surface modification reduced the membrane pore size to approximately 197 nm, significantly smaller than the typical diameter of Gram-negative bacteria (~270 nm). The combined effects of electrostatic repulsion and size exclusion resulted in complete (100%) bacterial removal.

However, although membranes with smaller and more uniform pore sizes enhance bacterial retention efficiency, they often result in a significant decline in flux. Furthermore, irregular or poorly connected pores can increase fluid resistance, further limiting treatment capacity. To address these issues, researchers have developed innovative membrane structures that maintain high flux while simultaneously enhancing filtration efficiency [6,11,12]. For instance, asymmetric membrane structures incorporate a dense filtration layer on one side for fine-particle retention, whereas a thicker porous support layer provides mechanical strength and facilitates fluid flow. This design enables the efficient removal of bacteria and viruses under high-flux conditions while minimizing pollutant buildup on the membrane surface [13,14]. Wickramasinghe et al. conducted filtration experiments with minute virus of mice (MVM) and bovine serum albumin (BSA) using DV20 asymmetric membranes, achieving an MVW retention of up to 99% and BSA retention of over 90%, significantly outperforming Omega300 symmetric membranes [15]. This structural design optimizes pore distribution, allowing effective retention of microorganisms of various sizes while avoiding excessive pressure differentials that could cause membrane damage or premature aging.

In addition to these applications, membrane technology is also used for the isolation of cell [16,17,18,19], and other particles [20,21,22,23]. Marcati et al. [24] investigated a two-step ultrafiltration process for the purification of B-phycoerythrin from red microalgal cells. An ultrafiltration membrane with a molecular weight cutoff of 300,000 Da was first used to separate high-molecular-weight polysaccharides, followed by a 100,000 Da membrane to further purify the target protein. This process successfully achieved a B-phycoerythrin purity of 48% with a purity index of 2.3. Elcik et al. [25] conducted a systematic study on membranes with different pore sizes and materials, finding that a membrane with a pore size of 0.45 µm performed well in terms of cell concentration and water flux but was prone to fouling by low molecular weight organic compounds. In contrast, membranes with a smaller pore size of 0.1 µm were more effective at retaining small microalgae cells; however, the reduced pore size rendered them more susceptible to irreversible fouling, resulting in a rapid decline in filtration flux. Zhang et al. [26] modeled the tangential flow filtration process and systematically studied the effects of transmembrane pressure (TMP) and tangential flow rate on filtration efficiency and membrane fouling. Their research demonstrated that optimizing the TMP and flow rate can significantly improve microalgae cell concentration efficiency and reduce membrane fouling issues.

During membrane filtration, when bacteria contact the membrane pores, the pressure differential across the membrane induces bacterial deformation, mainly because of the flexibility of the cell wall. If the pressure is sufficient, the bacteria elongate, thin out, and eventually pass through the pore [27]. As a distinct type of soft material, microgels exhibit responsiveness, reversible changes in volume and shape, and relatively uniform spherical structures, highlighting their unique potential applications in the biomedical field [28,29,30,31]. Because of their size variability, microgels are more advantageous than traditional rigid spheres, rendering them ideal model systems [32,33]. The Wessling et al. pioneered the use of microgels as soft model colloids to study separation and contamination in membrane filtration processes [34,35,36]. Previous studies have shown that microgels, as hydrated and deformable soft colloids, exhibit transport and adhesion behaviors comparable to those of bacteria, both governed by hydrodynamic and surface interactions. In both systems, retention and attachment are dominated by van der Waals, electrostatic, and steric forces. The inherent deformability of microgels also enables them to mimic the elongation and squeezing behavior of bacteria during pore passage [37]. Furthermore, microgels can be synthesized with precise control over their size and surface functionalization, providing a versatile platform for investigating the effects of membrane pore size and surface chemical properties on bacterial retention.

Herein, microgels with precisely controlled sizes (294, 362, 431, and 517 nm) were prepared to simulate the filtration behavior of bacterial suspensions through membranes. Two typical microporous membranes with similar average pore sizes but different pore structures were selected to determine the effect of membrane topology on dead-end filtration efficiency. Moreover, using microgels as probes, this study provides intuitive visual data on the behavior of microgels within membrane structures [38,39,40]. The experimental results and filtration model analysis offer a deeper understanding of media filtration and promising perspectives for the design and optimization of bacterial filtration membranes.

## 2. Materials and Methods

### 2.1. Materials

N-isopropylacrylamide (NIPAM, 99%), methyl acrylic acid (MAA, 99%), N, N′ -methylenebis (acrylamide) (BisAM, 99%), potassium persulfate (KPS, ≥99%) and sodium dodecyl sulfate (SDS, ≥99%) were purchased from Aladdin Co. Ltd. (Shanghai, China). Chloroplatinic acid hexahydrate (>99.9%) was purchased from Energy Chemical Co., Ltd. (Shanghai, China). Sodium hydroxide (NaOH, 96%) and hydrochloric acid (36–38%) were purchased from the Hangzhou Xiaoshan Chemical Reagent Factory (Hangzhou, China). NIPAM was purified by recrystallization and MAA was purified using an inhibitor remover column. All other chemicals were used as received. A PES membrane (trade name HPWP04700) with a highly asymmetric textile structure was purchased from Merck Millipore (Burlington, MA, USA). A PES membrane (trade name MLS04504750N) with a highly symmetric textile structure was purchased from Anow (Hangzhou, China). Deionized (DI) water was produced using an Aquelix5 apparatus (Millipore). In the following section, HPWP and MLSN refer to the structurally asymmetric and symmetric PES membranes, respectively.

### 2.2. Synthesis of Hybrid Micro-Hydrogels PNM-Ag-n (n = 1–4)

The functional hydrophilic monomer NIPAM and MAA, crosslinker BisAM and emulsifier SDS were dissolved in DI water and initiated with KPS at 70 °C under a N_2_ atmosphere. After polymerization, the pH stimuli responsible P(NIPAM-co-MAA) microgels (PNM) were collected by centrifugation and subsequently lyophilized. Four microhydrogels with different diameters (PNM1-4) were successfully synthesized by varying the amount of deionized water in the synthetic formulation. Furthermore, to enable the visible detection of microgels on and within the membrane, yellow hybrid P (NIPAM-co-MAA)-Ag microhydrogels (PNM-Ag-n (*n* = 1–4)) were prepared using an in situ reduction method [41].

### 2.3. Filtration of Microgel Suspension

**Figure 1:**
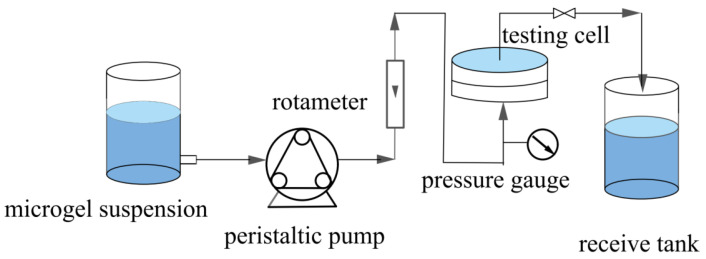
Schematic of the constant-flux filtration setup.

The microgels were dispersed in water by ultrasonication for 30 min to obtain a microgel suspension with a specified solid content (5–90 mg/L). The pH of the suspension was then adjusted to 3. Subsequently, a specified volume of microgel suspensions was subjected to constant-flux and dead-end filtration through two different types of membranes, HPWP and MLSN, as detailed in Table 1; the filtration was performed in a steel module at 20 °C. Figure 1 shows a schematic of the filtration setup. The variation in TMP during operation was recorded using gauges. The retention rate (*R*) of the microgel membrane was calculated using Equation (1):

(1) $$R=\frac{m}{m_{0}}\times 100,$$

where *m* is the weight gain of the membrane after filtration and *m*_0_ is the overall mass of the microgel in the suspension.

### 2.4. Characterization

The morphology of the microgel sample was examined using a JEM-1400 transmission electron microscope (TEM, JEOL, Japan). The microgel powder was first ultrasonically dispersed in deionized water to obtain a uniform suspension. A small amount of the dispersion was then dropped onto a copper grid and dried at room temperature. The largest effective pore size of the nascent membranes were determined based on the bubble point method using a pore size analyzer (BSD-PB, BSD Instruments, Beijing, China). Before testing, the membranes were soaked in anhydrous ethanol. The hydrodynamic diameter and distribution of the hybrid microgels at pH = 3 were measured by dynamic light scattering (DLS, Zetasizer Nano ZS90, Malvern, UK) at 20 °C. The weight gain of the membranes was determined using a precision electronic balance (FA2104N, Techcomp, Hong Kong, China). Parallel tests were performed five times for each sample. The morphologies of the membranes were examined using scanning electron microscopy (SEM, S-4700, Hitachi, Japan). To obtain well-defined cross-sectional structures, the samples were frozen in liquid nitrogen and fractured. All samples were subjected to gold sputtering for 10 min.

## 3. Results and Discussion

### 3.1. Synthesis and Characterization of Microgels

**Figure 2:**
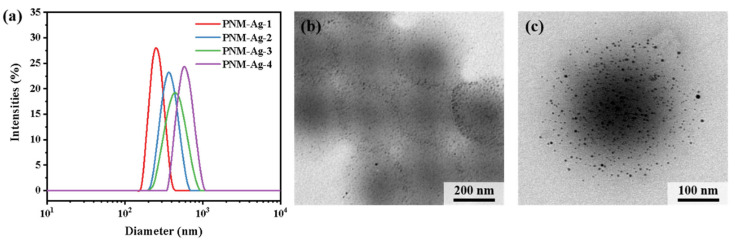
(**a**) Size distribution of microgels PNM-Ag-n (*n* = 1–4). (**b**,**c**) TEM images of PNM-Ag-2.

The hybrid microgels PNM-Ag-n (*n* = 1–4) were synthesized via precipitation polymerization followed by in situ reduction. To regulate the particle size, the monomer concentration during the polymerization stage was systematically varied from 1.0 wt% to 1.9 wt%. The particle sizes and distributions of the resulting microgels were characterized by DLS measurements (Figure 2a). The average hydrodynamic diameters of PNM-Ag-1 to PNM-Ag-4 were 294, 362, 431 and 517 nm, respectively, with corresponding particle dispersion indices (PDI) of 0.025, 0.032, 0.085, and 0.114 (Table 2). All microgels exhibited relatively narrow, approximately normal size distributions, making them suitable as standard samples for investigating dynamic filtration processes. Figure 2b,c present TEM images of the representative microgel PNM-Ag-2. The microgel particles exhibit a spherical morphology and uniform size. Dark spots visible within the microgels correspond to embedded Ag nanoparticles. These nanoparticles display surface plasmon resonance, which absorbs violet light near 400 nm (Figure S1) and produces a complementary yellow color. In following filtration experiments, the spatial distribution and intensity of this yellow coloration provide preliminary insight into the retention sites of microgels within the membranes.

### 3.2. Filtration of Microgel Suspensions in MLSN and HPWP Membranes

**Figure 3:**
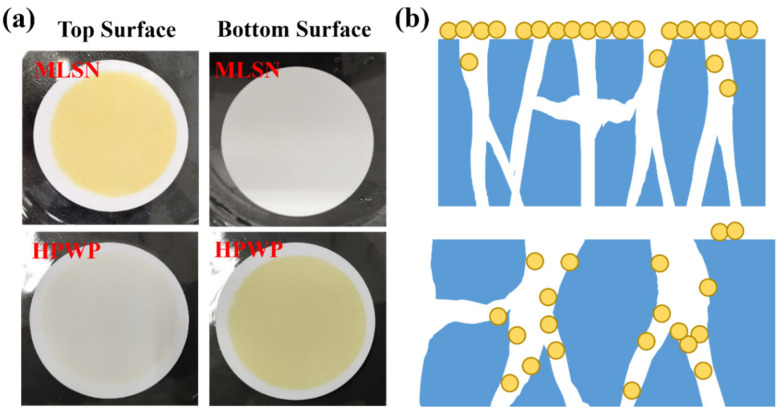
(**a**) Appearance of the membranes after filtration of PNM-Ag-2 suspensions. (**b**) Schematic of the proposed retention mechanisms for the MLSN (**top**) and HPWP membranes (**bottom**).

A PNM-Ag-2 microgel suspension (10 mg/L) was employed as the feed solution to evaluate the filtration behavior of the symmetric and asymmetric HPWP membranes (flow rate: 190 mL/min). Upon complete consumption of the feed solution (0.5 L), the top surface of the MLSN membrane displayed a yellow coloration, whereas the bottom surface remained white (Figure 3a), indicating that the PNM-Ag-2 microgels were predominantly retained near the upper surface and did not permeate through the microporous structure. This observation suggests that the primary retention mechanism in the MLSN membrane was surface filtration (Figure 3b). For the HPWP membrane, the filtrate exhibited a light-yellow hue, implying that some PNM-Ag-2 microgels partially passed through the membrane. This can be attributed to the microgel particle size (362 nm) being smaller than the pore throat diameter of the HPWP membrane. After filtration, both the top and bottom surfaces of the HPWP membrane appeared yellow, with the bottom surface exhibiting a darker coloration, indicating that microgel retention primarily occurred within the deeper pores of the HPWP membrane.

**Figure 4:**
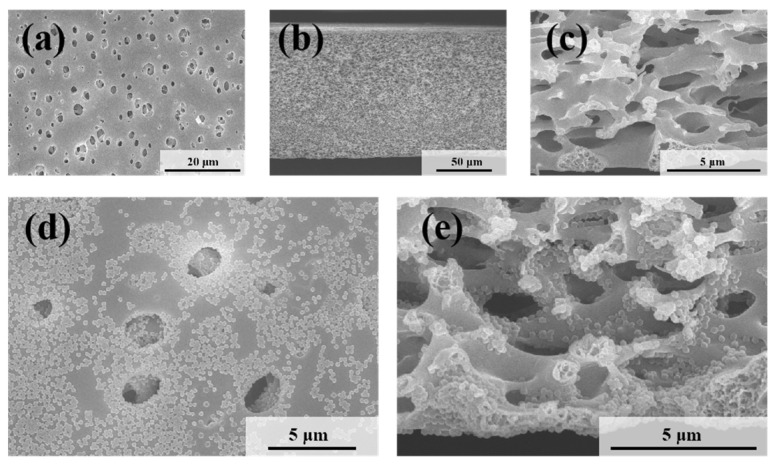
SEM images of (**a**) the top surface and (**b**,**c**) the cross-section of the nascent MLSN membrane; SEM images of (**d**) the top surface and (**e**) the cross-section of the MLSN membrane after filtration of PNM-Ag-2 suspensions.

The microscopic morphologies of the original symmetric microporous membrane and the MLSN membrane after filtration were examined using SEM. The surface pore size ranged from approximately 0.5 to 2.5 μm, and the pores exhibited an hourglass shape across the cross-section (Figure 4a–c and Figure S2). Larger pores were present near the upper and lower surfaces, whereas smaller pores were observed in the middle of the membrane. The bubble-point pore size was determined to be 0.46 μm (Figure S3). Owing to the low pore-opening ratio on the membrane surface, the microgels tended to bridge the pore openings, resulting in partial blockage (Figure 4d). Substantial accumulation of microgels was observed on the upper surface of the membrane. Moreover, some microgels penetrated the membrane channels and became adsorbed or trapped along the pore walls (Figure 4e) because the diameter of the contracted PNM-Ag-2 microgels was smaller than the surface pore size of the MLSN membrane. The hybrid microgels were primarily concentrated within a depth of approximately 0–10 μm near the upper surface of the membrane. The distribution of the hybrid microgels observed in the SEM images closely aligned with a previously proposed hypothesis. The smaller particle sizes compared with those obtained from DLS measurements are attributed to the drying of the microgel samples during SEM preparation.

**Figure 5:**
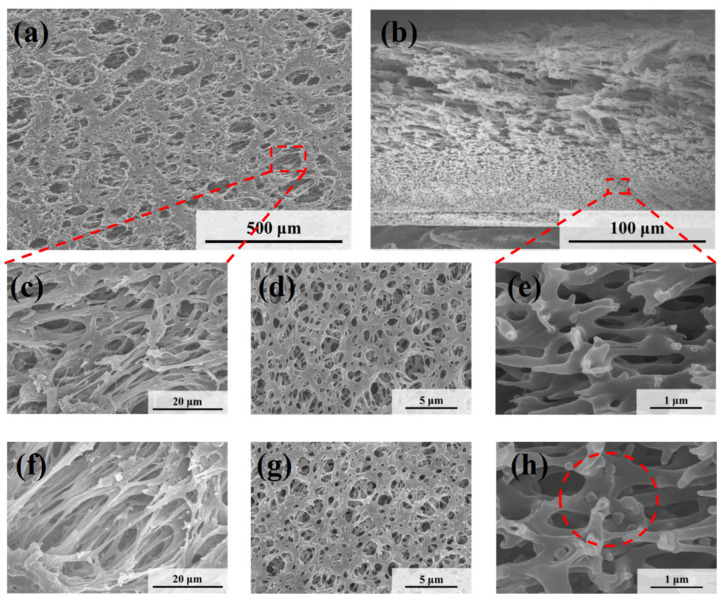
SEM images of (**a**,**c**) the top surface, (**b**,**e**) the cross-section and (**d**) the bottom surface of the nascent HPWP membrane; SEM images of (**f**) the top surface, (**g**) the bottom surface, and (**h**) the cross-section of the HPWP membrane after filtration of PNM-Ag-2 suspensions.

Figure 5 shows the microscopic morphologies of the HPWP membranes before and after filtration. Compared to the MLSN membrane, the HPWP membrane exhibits significantly higher surface porosity and larger pore sizes, ranging from approximately 5 to 20 μm. The pore sizes gradually decreased from top to bottom along the cross-section, indicating that the HPWP membrane had a highly asymmetric gradient pore structure. The throat pore size of the membrane measured using the bubble-point method was 0.48 μm (Figure S4). It should be noted that the bubble-point method measures the largest effective pore size at the pore throats, rather than the surface macropores of the asymmetric structure. After filtration, the PNM-2 microgels were primarily located in the pore throats of the HPWP membrane, with a relatively low distribution density. Energy-dispersive spectroscopy (EDS) analysis of the membrane cross-section revealed the co-existence of N and Ag elements, further confirming the entrapment of microgels within the membrane (Figure S5). Moreover, a small amount of the microgel was adsorbed on both the upper and lower surfaces of the membrane. The above experiments demonstrate that the highly asymmetric pore structure facilitated the entry of the hybrid microgels into the interior of the microporous membrane, where they became trapped. The trapping process primarily followed a deep-filtration mechanism. This characteristic enhances the effective filtration area of the membrane and increases its overall processing capacity.

### 3.3. Filtration Characteristics of Microgels of Different Sizes in HPWP (Asymmetric) Membrane

**Figure 6:**
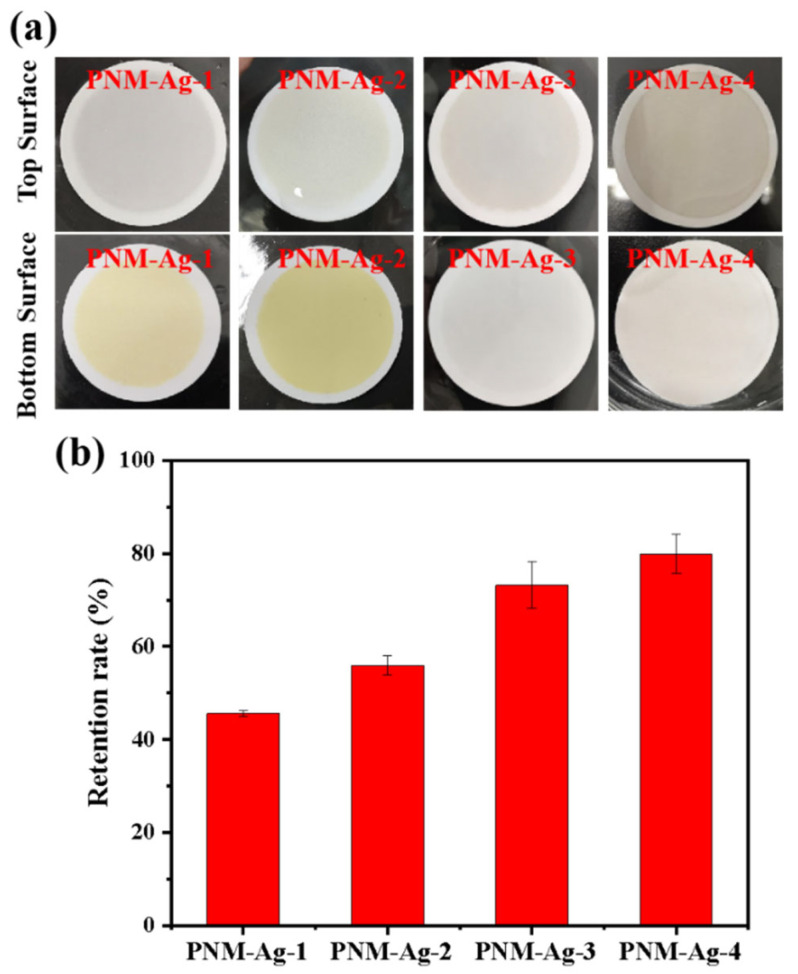
(**a**) Appearance of the HPWP membranes after filtration with microgel suspensions of different particle sizes; (**b**) Retention rate for microgel particles of different sizes by the HPWP membrane. Error bars represent standard deviations based on three independent measurements.

Submicron microgels with average particle sizes of 294, 362, 431, and 517 nm were used to further investigate the filtration characteristics of the HPWP membranes (asymmetric) at constant rates. The filtration process involved passing 1 L of microgel suspension (10 mg/L) through the HPWP membrane, followed by washing with a 0.01 M NaOH solution. For microgels with particle sizes of 294 nm (PNM-Ag-1) and 362 nm (PNM-Ag-2), the upper surface of the HPWP membrane showed minimal color change, whereas the lower surface appeared buff. This indicated that the microgels in the membrane were primarily retained near the bottom surface (Figure 6a). The collected filtrate was light yellow, suggesting that the PNM-Ag-1 and PNM-Ag-2 microgels partially passed through the HPWP membrane. In contrast, after filtering the microgels with particle sizes of 431 nm (PNM-Ag-3) and 517 nm (PNM-Ag-4), the bottom surface of the HPWP membrane showed almost no coloration, whereas the upper surface displayed a significant color change. The retention rates of microgels of different sizes filtered through the HPWP membrane are shown in Figure 6b. As the size of the microgels increased, their membrane retention rate gradually increased. Notably, microgels with average sizes smaller than the pore throat diameter of the membrane (PNM-Ag-1 and PNM-Ag-2) formed minimal “cake layers” on the HPWP membrane surface and were instead trapped at the pore throats. By comparison, microgels with average sizes comparable to or larger than the pore throat diameter of the HPWP membrane (PNM-Ag-3 and PNM-Ag-4) partially penetrated the membrane pores and also accumulated on the surface. This behavior can be attributed to the gradual decrease in the pore size from top to bottom along the cross-sectional direction of the HPWP membrane. Microgels with average sizes of 431 and 517 nm were impeded and trapped within specific locations of the membrane pores, leading to pore blockage caused by mechanical action or bridging effects. Consequently, the accumulation of microgels within membrane pores leads to pore blocking and the formation of a “cake layers” on the membrane surface.

### 3.4. Filtration Characteristics of Microgel Suspensions with Different Solid Content in the HPWP (Asymmetric) Membrane

**Figure 7:**
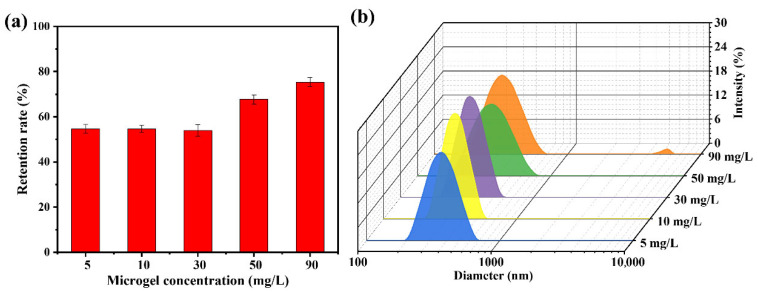
(**a**) Retention rate of the HPWP membrane toward PNM-Ag-2 microgel suspensions at different solid contents; (**b**) Particle diameter distributions of the PNM-Ag-2 microgel suspensions with different solid contents. Error bars represent standard deviations based on three independent measurements.

The solid content of the dispersion medium exhibited a strong correlation with the dispersion state of the particles, probability of collisions between the particles and the membrane, and interparticle collisions during filtration. These factors collectively influence the microgel retention efficiency of microporous membranes under dynamic filtration conditions. As shown in Figure 7a, when the solids content of the microgel suspension was below 30 mg/L, the retention rate remained stable at approximately 54%, with minimal variation as the concentration increased. In addition, negligible “cake layers” formation was observed on the membrane surface. Increasing the solid content to 50 mg/L enhanced the microgel retention rate, yet concurrently induced a cake layer on the membrane surface. Further elevation of the solids content to 90 mg/L resulted in a retention rate of 75.3% and pronounced accumulation of the cake layer. To elucidate the underlying causes, the dispersion states of the microgel particles in suspensions with varying solid contents were characterized using DLS (Figure 7b). When the solids content was below 30 mg/L, the average particle size was approximately 360 nm with a low PDI of approximately 0.08, indicating that the microgels primarily existed as individual particles without significant aggregation. As the solids content increased to 50 mg/L, the particle size distribution broadened, and the average particle size increased to approximately 380 nm. Notably, particles in the 600–700 nm range accounted for 6.4% of the total population, suggesting that a higher solid content increases interparticle collisions and aggregation. Aggregated microgels were more prone to retention due to bridging effects or mechanical entrapment. A further increase in the solids content to 90 mg/L resulted in a similarly broad particle size distribution, with the appearance of significantly larger species (4800–5500 nm), which constituted approximately 1.6% of the total microgel population, thereby further enhancing retention.

### 3.5. Membrane Blockage Mechanism in the Filtration Process

**Figure 8:**
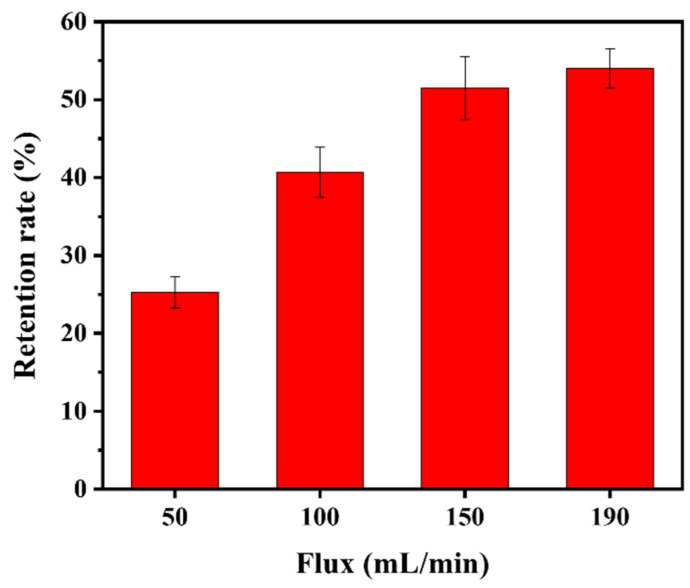
Retention rate of the HPWP membrane for PNM-Ag-2 at different fluxes. Error bars represent standard deviations based on three independent measurements.

The retention rate of the PNM-Ag-2 microgels (30 mg/L) by the HPWP membrane at four different fluxes is illustrated in Figure 8. As the flux increased from 50 to 150 mL/min, the retention rate increased rapidly from 25.3% to 51.5%, respectively. However, when the filtration rate was further increased to 190 mL/min, the retention rate showed only a slight increase, with the rate of improvement slowing. No “cake layers” was observed on the surface of the HPWP membrane at any of the filtration rates tested. Owing to the maximum flux limitation of the peristaltic pumps used, tests were not performed at larger filtration rates. These phenomena can be explained as follows: when the filtration rate increases, the microgels are more likely to deviate from the liquid flow path owing to inertia, leading to more frequent collisions with the pore walls. This increased probability of inertial collisions resulted in greater adsorption of microgels onto the pore walls. In addition, an increase in filtration rate increases the probability of particle bridging.

**Figure 9:**
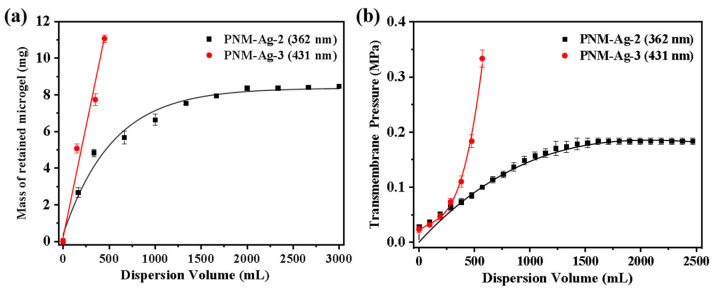
Effect of microgel suspension filtration volume on (**a**) the mass of retained microgels and (**b**) transmembrane pressure. The effective membrane area is 12.56 cm^2^. Error bars represent standard deviations based on three independent measurements.

Additional experiments were conducted by systematically varying the microgel suspension filtration volume to determine whether adsorption or particle bridging was the predominant mechanism. For PNM-Ag-2, its retention by the HPWP membrane can be divided into three stages, as shown in Figure 9a: (1) when the consumption of the microgel suspension was less than or equal to 330 mL, the retention of the microgel by the membrane increases rapidly with increasing consumption of the suspension; (2) when the microgel suspension processed ranged between 330 and 1660 mL, the retention rate of the membrane for the microgel gradually decreased, and the growth rate of the membrane mass reduced; (3) when the consumption of the microgel suspension exceeded 1660 mL, the membrane reaches its maximum retention capacity, stabilizing at approximately 8.4 mg. This outcome indicated a dynamic equilibrium between microgel retention and loss by the membrane. At a constant filtration rate, the trend in the transmembrane pressure change was highly consistent with the mass variation of the membrane. It increased steadily in the initial stage, slowed during the intermediate stage, and remained relatively stable during the final stage. During the filtration of PNM-Ag-3 (431 nm), the rejection of the microgels by the HPWP membrane was directly proportional to the consumption of the suspension, with a retention rate of approximately 81%. In this process, the transmembrane pressure increased exponentially as suspension consumption increased (Figure 9b).

Two distinct membrane fouling processes during filtration were analyzed using classical blocking and adsorption models. As particles are deposited during the filtration process, the membrane pores gradually become blocked, resulting in increased transmembrane resistance. Hermans and Brebee proposed four blocking models based on different predominant membrane fouling: (1) complete blocking, (2) standard blocking, (3) intermediate blocking, and (4) cake filtration [42]. Hermia et al. proposed a general form of these four membrane-blockage models for constant-rate filtration (Equation (2)) [43].

(2) $$\frac{d(∆v)}{dv}={k\left(∆p\right)}^{i},$$

where Δ*P* is the transmembrane pressure, *V* is the filtrate volume, *k* is the resistance coefficient, and *i* is the blockage index.

**Figure 10:**
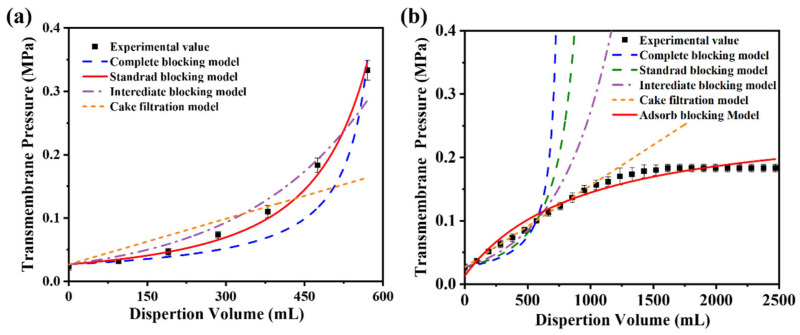
Comparison of simulated and experimental values of the blocking model during constant-velocity filtration of microgel suspensions with average particle sizes of (**a**) 431 nm and (**b**) 362 nm. The effective membrane area is 12.56 cm^2^. Error bars represent standard deviations based on three independent measurements.

Figure 10a presents the simulated and experimental transmembrane resistance values as a function of the filtrate volume during the constant filtration of microgels with a diameter of 431 nm. The results demonstrate that the standard blocking model aligns closely with the experimental data, achieving a correlation coefficient (R^2^) of 0.99. These findings suggest that the primary factor contributing to the increase in transmembrane resistance during filtration is a gradual reduction in the effective pore size of the membrane. The HPWP membrane exhibits an asymmetric pore structure. When microgel particles enter the membrane pores, they are retained through adsorption and bridging at specific locations within the membrane. Bridging halts the downward penetration of microgel particles, causing them to adsorb onto the pore walls of larger membranes, thereby reducing the effective pore size of the membrane and increasing its transmembrane resistance.

For constant-flux filtration using microgels with a particle diameter of 362 nm as standard particles, the fitting results of the four classical models showed significant deviations from the experimental values (Figure 10b). The possible reasons for this phenomenon are as follows. (1) The HPWP membrane has an asymmetric pore structure with a larger surface pore size. Owing to the smaller size of the microgel particles compared with the pore size, the particles cannot be effectively retained through surface filtration, nor can they entirely block the membrane pores. (2) The microgels exhibited partial swelling in water and deformed to pass through the membrane pores under increased pressure. This behavior deviates from the predictions of the standard blocking model. (3) According to the experimental observations, no filter “cake layers” appeared on the membrane surface during filtration. The microgels were primarily trapped through deep filtration and did not align with the filter cake filtration model. These factors contributed to the significant deviation between the fitting results of the classical models and the experimental values for this filtration process. Assuming that only the adsorption resistance and new membrane resistance are considered, while factors such as membrane pore clogging, “cake layers” formation, and concentration polarization are neglected, the relationship between the transmembrane resistance and dispersant consumption during constant-velocity filtration can be described by the adsorption model (Equation (3)) [44]:

(3) $$∆p=\frac{v}{a+bv}+∆p_{0},$$

where Δ*P*_0_ represents the transmembrane resistance (new membrane resistance) during pure water filtration, while *a* and *b* are model parameters obtained through regression analysis of experimental values. Compared to the four classical models, the adsorption model showed a higher degree of agreement between the simulated results and experimental values, with a correlation coefficient R^2^ of 0.98. Despite the oversimplification of the filtration process in the adsorption model, the results were sufficient to demonstrate that the PNM-Ag-2 microgels (362 nm) were primarily retained through adsorption. When the consumption of the microgel suspension reaches a certain threshold, an “adsorption–desorption” equilibrium is established, and the membrane’s retention capacity for microgels reaches its maximum.

The difference in transmembrane pressure models between the 431 nm and 362 nm microgels reflects the interplay between particle size and the asymmetric pore structure of the HPWP membrane. The 431 nm microgels, comparable in size to the pore throats, are primarily retained through adsorption and bridging, narrowing the pores and increasing resistance, consistent with the standard blocking model (R^2^ = 0.99). In contrast, the smaller 362 nm microgels can deform and swell under pressure, partially penetrating deeper membrane layers. Their retention is dominated by deep adsorption rather than pore blockage, in line with the adsorption model and the absence of a surface “cake layers”. This shift from blocking to adsorption-dominated behavior can be preliminary predicted from the relative sizes of the microgels and membrane pores: when particle diameters approach the pore throat, blocking dominates; when smaller, deep adsorption prevails.

## 4. Conclusions

In this study, microgel particles with controllable sizes were prepared as bacterial analogs to evaluate the filtration characteristics of microporous PES membranes (nominal pore size 0.45 μm) with symmetric (MLSN) and asymmetric (HPWP) architectures. A comparative analysis revealed that the asymmetric HPWP membrane, characterized by an open upstream surface and graded pore structure, exhibited a significantly larger effective filtration area than its symmetric counterpart (MLSN). For the HPWP, smaller microgels (294 nm and 362 nm) were primarily retained at the pore throats, whereas larger microgels (431 nm and 517 nm) partially penetrated the pores and accumulated on the surface. The adsorption model indicated that the 362 nm microgels were mainly retained by the membranes through adsorption mechanisms, while the 431 nm microgels conformed to the standard blockage model. This study demonstrated that the size of the microgels significantly affected their retention behavior in membranes, especially in asymmetric structures. These findings not only provided important scientific insights for the design of bacterial filtration membranes but also emphasized the importance of optimizing membrane selection and operational conditions in the bio-pharmaceutical process to enhance retention efficiency and reduce clogging. The use of microgels as substitutes for bacteria further demonstrated their potential for simulating bacterial filtration behavior, offering an effective tool for studying bacterial filtration under safe and controlled experimental conditions.

## Figures and Tables

**Table 1:** Summary of membrane information.

Membrane	Material	Structure	Nominal Pore Size
HPWP	PES	Asymmetrical	0.45 μm
MLSN	PES	Symmetrical	0.45 μm

**Table 2:** Summary of PNM-Ag-n (*n* = 1–4) characterizations (pH = 3, T = 20 °C).

Microgel Suspension	Average Diameters (nm)	PDI
PNM-Ag-1	294	0.025
PNM-Ag-2	362	0.032
PNM-Ag-3	431	0.085
PNM-Ag-4	517	0.114

## Data Availability

Data is contained within the article or Supplementary Material.

## Supplementary Materials

The following supporting information can be downloaded at: https://www.mdpi.com/article/10.3390/polym17212958/s1, Figure S1: UV-vis absorption spectra of PNM-Ag-2; Figure S2: (a) Top, (b) middle, and (c) bottom regions of the cross-section SEM images of the symmetric MLSN membrane; Figure S3: Pore size of the nascent membrane with symmetrical structure; Figure S4: Pore size of the nascent membrane with asymmetrical structure; Figure S5: Energy dispersive spectroscopy of the HPWP membrane cross-section after filtration with PNM-Ag-2 suspensions.
