# Supplementary material for: Detection of Filtration Characteristics of Nontraditional Asymmetric Microporous Membranes Using Size-Controllable Micro-Hydrogel
Source: Polymers (Basel). 2025 Nov 6;17(21):2958. doi: 10.3390/polym17212958 (PMC12608552; doi:10.3390/polym17212958)
Supplement: Supplementary file 1 [file polymers-17-02958-s001.zip › polymers-3935352-supplementary.pdf]

Supplementary Materials

Detection of filtration characteristics of nontraditional asymmetric microporous membranes using size-controllable micro-hydrogel

*Hao Zhang, Tiantian Zhu, Yushan Zheng, Weiheng Liu, Tangxin Zhang, Yuhua Mao, Jiayuan Wang, Lingyu Zhu, Cheng Xu and Jianli Wang**

State Key Laboratory of Advanced Separation Membrane Materials, State Key Laboratory of Green Chemical Synthesis and Conversion, Zhejiang Province Key Laboratory of Biofuel, College of Chemical Engineering, Zhejiang University of Technology, Hangzhou 310014, PR China
E-mail: wangjl@zjut.edu.cn

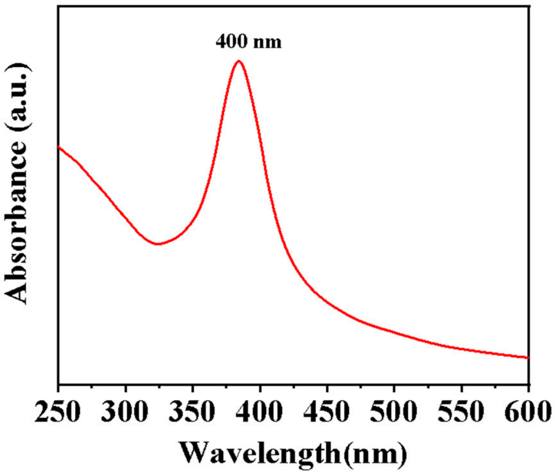

Figure S1. UV-vis absorption spectra of PNM-Ag-2.

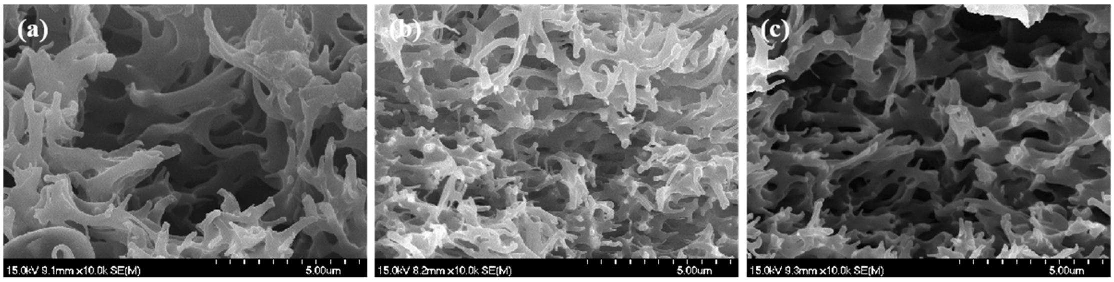

Figure S2. (a) Top, (b) middle, and (c) bottom regions of the cross-section SEM images of the symmetric MLSN membrane.

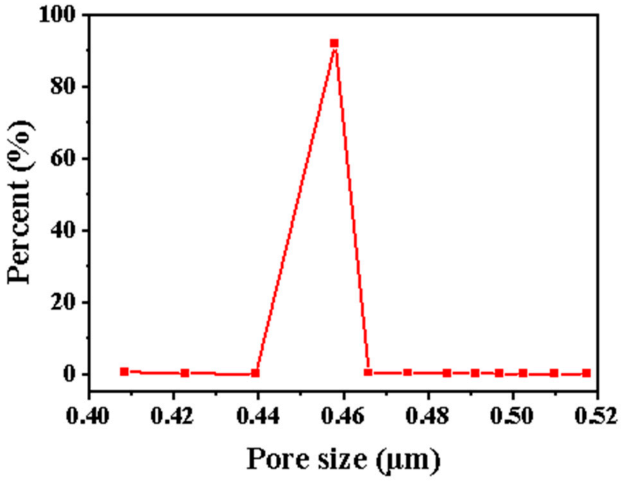

Figure S3. Pore size of the nascent membrane with symmetrical structure.

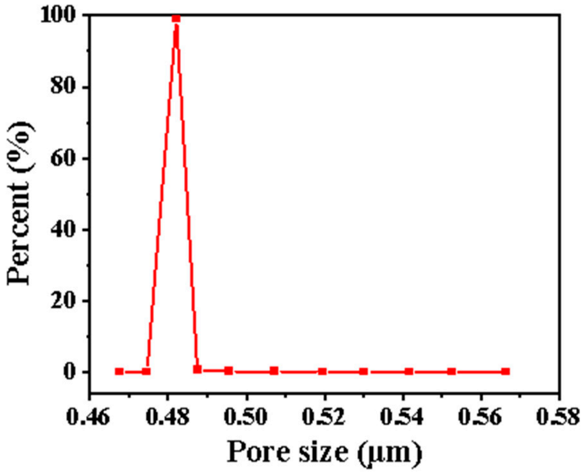

Figure S4. Pore size of the nascent membrane with asymmetrical structure.

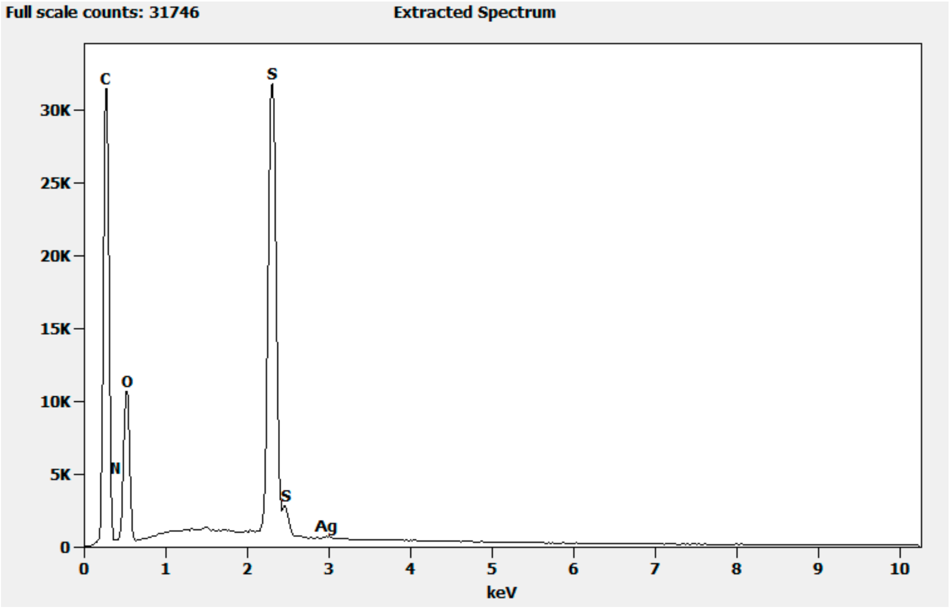

Figure S5. Energy dispersive spectroscopy of the HPWP membrane cross-section after filtration with PNM-Ag-2 suspensions.
